# Nitrogen and Phosphorus Removal from Wastewater Treatment Plant Effluent via Bacterial Sulfate Reduction in an Anoxic Bioreactor Packed with Wood and Iron

**DOI:** 10.3390/ijerph110909835

**Published:** 2014-09-22

**Authors:** Takahiro Yamashita, Ryoko Yamamoto-Ikemoto

**Affiliations:** 1 NARO Institute of Livestock and Grassland Science, National Agriculture and Food Research Organization, 2 Ikenodai, Tsukuba, Ibaraki 305-0901, Japan; E-Mail: yamatktk@affrc.go.jp; 2 Institute of Science and Engineering, Kanazawa University, Kakuma-machi, Kanazawa, Ishikawa 920-1192, Japan

**Keywords:** denitrification, iron, phosphate removal, sewage treatment plant effluent, wood

## Abstract

We investigated the removal of nitrogen and phosphate from the effluent of a sewage treatment plant over a long-term operation in bioreactors packed with different combinations of wood and iron, with a trickling filter packed with foam ceramics for nitrification. The average nitrification rate in the trickling filter was 0.17 kg N/m^3^∙day and remained at 0.11 kg N/m^3^∙day even when the water temperature was below 15 °C. The denitrification and phosphate removal rates in the bioreactor packed with aspen wood and iron were higher than those in the bioreactor packed with cedar chips and iron. The bioreactor packed with aspen wood and iron continued to remove nitrate and phosphate for >1200 days of operation. The nitrate removal activity of a biofilm attached to the aspen wood from the bioreactor after 784 days of operation was 0.42 g NO_3_-N/kg dry weight wood∙ day. There was no increase in the amount of dissolved organic matter in the outflow from the bioreactors.

## 1. Introduction

The removal of nitrogen and phosphorus from wastewater has become an emerging worldwide concern because these compounds cause eutrophication in natural water. Moreover, nitrate is a risk to human health, especially as a possible cause of infant methaemoglobinaemia [1]. An activated sludge process is commonly used in wastewater treatment, but it is often the case that the effluent from wastewater treatment plants has remaining phosphorus and nitrogen in the form of ammonium and/or nitrate. A post-treatment process is therefore required to remove nitrogen and phosphorus from the effluent. However, because such effluents contain only small amounts of organic compounds, a carbon source must be added to remove the nitrogen.

The conventional methods for post-treatment denitrification often employ methanol for the removal of nitrate [2]. Although methanol does achieve a high rate of denitrification, there are concerns about the risks posed by treatment-plant outflow containing excessive amounts of organic carbon and its flammability [3]. In practice, wastewater treatment managers hope to minimize post-treatment operation and maintenance costs. Therefore, post-treatment technologies that will enable the use of waste materials such as municipal refuse, agro-industrial residues, and wood waste are desired. In a previous study, nitrate was successfully removal from synthetic wastewater in a bioreactor packed with wood as an organic carbon source under anoxic conditions. Interestingly, the denitrification efficiency was assumed to enhance sulfur denitrification via wood degradation by sulfate reduction [4].

Sulfate-reducing bacteria can use wood chips or animal manure as electron donors and carbon sources and then use various types of organic substances [5]. The sulfate-reducing bacteria *Desulfovibrio* sp. CMX have been used to remove nitrogen oxide (NO) in iron/ethylenediaminetetraacetic acid (FeEDTA) solutions [6]. Yücel *et al*. [7] reported that sulfate reduction (*i.e.*, sulfide production) occurs in degrading wood in marine environments. Sulfate-reducing bacteria seem to play an important role in nitrogen removal with wood. On the other hand, ferrous ions generated by iron polarization are known to combine with phosphate to produce vivianite and other ferrous phosphates, and thereby remove phosphorus [8]. Till *et al*. [9] demonstrated that steel wool was effective at removing nitrogen in autotrophic denitrifying bacteria. However, iron corrosion is enhanced under oxic conditions and under high nitrate conditions. For this reason, the addition of another electron donor is useful to support the corrosion resistance. We therefore hypothesized that a bioreactor packed with iron and wood could remove nitrogen and phosphorus simultaneously over a long-term operation. And indeed, our earlier study showed that both denitrification and phosphate removal occurred in a bioreactor filled with synthetic wastewater and packed with iron and wood and allowed to react over a long-term [10].

In the present study, we demonstrated that nitrogen and phosphorus were removed from the effluent of a sewage treatment plant using an anoxic bioreactor packed with wood and iron. We measured the nitrogen and phosphorus removal activity and then the sulfur denitrification activity; we used dissimilatory (bi)sulfite reductase gene-targeted nested polymerase chain reaction-denaturing gradient gel electrophoresis (PCR-DGGE) to analyze the sulfate-reducing bacteria growing inside the wood and predict the characteristics of sulfate reduction. Since most of the effluent was ammonium as a nitrogen source, a trickling filter packed with foam ceramics was used for nitrification. Foam ceramics are a type of porous media characterized by high water retention, and they contribute to biofilm growth for nitrifying bacteria. The reactor was configured as a trickling-filter type reactor in order to conserve energy for the oxygen supply by aeration.

## 2. Materials and Methods

### 2.1. Reactor and Experimental Procedure

**Figure 1:**
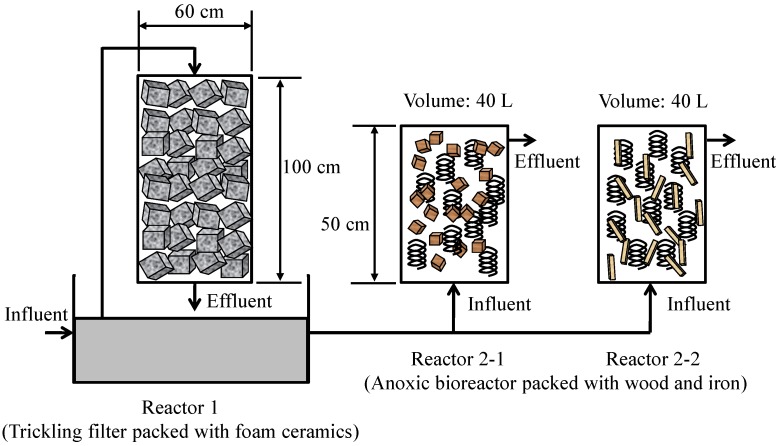
Experimental set-up. The influent of Reactor 1 was the effluent from the final sedimentation basin of the conventional activated sludge process of the treatment plant. The influent of Reactor 2 was the effluent of Reactor 1. Reactor 2-1 was packed with cedar chips and iron. Reactor 2-2 was packed with chopsticks waste (made of aspen wood) and iron.

Figure 1 shows the setup of the trickling filter and anoxic reactors used in this study. The reactors were set in a sewage treatment plant (STP) in Kanazawa, Japan and operated using the effluent from the final sedimentation basin of the conventional activated sludge process of the plant. The trickling filter packed with foam ceramics (Reactor 1) was used to convert NH_4_^+^-N to NO_3_^−^-N for nitrification under aerobic conditions. The foam ceramics were synthesized by a calcination treatment of diatomaceous earth using iron and steel slag as waste materials. The trickling filter was made of a round column (60 cm diameter, 100 cm high), and the total volume of the reactor was approximately 283 L. The reactor was fully packed with 5 × 5 × 5-cm cubes of the foam ceramics as a microbial supporting medium. The reactor was set up on a storage tank, and the effluent of the final sedimentation basin was supplied to the storage tank. The water in the storage tank trickled onto the reactor. The hydraulic retention time (HRT) of the reactor was 2.4 ± 1.8 h (mean ± SD). The recycle flow ratio was 8 ± 7.5 (mean ± SD).

The anoxic reactors were used to convert NO_3_^−^-N to N_2_ for denitrification and to remove phosphate. The total volume of the 50-cm-high reactor was 40 L. The effluent of Reactor 1 was fed to the bottom of the anoxic reactors (Reactors 2-1 and 2-2). Reactor 2-1 was packed with 3.8 kg of cedar chips (10–30 mm wide, 5 mm thick) and 1.6 kg of iron coils (S55C, 0.5–1 mm wide, 0.1–0.2 mm thick). Reactor 2-2 was packed with 3.8 kg of chopsticks waste and 1.6 kg of iron coils. The chopsticks were made of aspen wood and had been used in restaurants and then discarded. The used chopsticks were rinsed with tap water and cut to approximately 100 mm high, 7 mm wide and 4 mm thick.

The return sludge collected from the STP was seeded in the reactors. Before the start of the experiment, Reactors 2-1 and 2-2 were operated under sulfidogenic conditions, in which 10 L of K_2_SO_4_ (300 mg/L of sulfate)-supplemented effluent from the final sedimentation basin was used as the medium. The medium was added to Reactors 2-1 and 2-2 once a week for 82 days. The HRTs of Reactors 2-1 and 2-2 were set at 6 to 24 h, depending on the nitrogen and phosphorus removal rates. Table 1 shows the HRTs of Reactors 2-1 and 2-2 during the present study’s operational period. The influents and effluents of Reactors 1, 2-1 and 2-2 were collected once a week, at which time we measured the pH and the concentrations of sulfate, nitrate, nitrite, ammonium, organic acids, bicarbonate, total organic carbon (TOC), and total nitrogen (TN).

**Table 1:** The hydraulic retention times (HRTs) of Reactors 2-1 and 2-2 during the operational period.

Operational Period (Days)	HRT (Hours)
0–150	24
151–259	18
260–343	12
344–440	6
441–557	12
558–1224	24

### 2.2. Batch Experiments to Analyze the Sulfate Reduction and Denitrification Activities with the Use of Wood in Reactors 2-1 and 2-2

The batch experimental conditions are shown in Table 2. Experiments B1 and B2 were conducted under anaerobic conditions, and Experiments S1 and S2 were conducted under sulfidogenic conditions. Experiments N1 and N2 were conducted under anoxic (denitrification) conditions, and Experiments TN1 and TN2 were conducted under anoxic (sulfur denitrification) conditions. Some pieces of wood with biofilm were taken out of the anoxic reactors (Reactors 2-1 and 2-2) after 593, 784, 1104 and 1224 days of operation.

The sulfate reduction and denitrification activities in the biofilm inside the wood were measured by batch experiments under stationary conditions according to the following procedure: 25-g wet weight of cedar chips or used chopsticks was put into a 100-mL bottle, and then 90 mL of sterilized effluent from the STP supplemented with 181 mg/L of K_2_SO_4_, 137 mg/L of NaNO_3_ or 250 mg/L of Na_2_S_2_O_3_∙5H_2_O that was purged with nitrogen gas was added to the bottle, and the bottle was kept stationary at 20 °C. The mixed liquor was taken out of the bottle at 24-h intervals, and the concentrations of sulfate, nitrate, nitrite, acetate, propionate and bicarbonate in the filtered sample were measured. After these experiments the wood materials, including their biofilms, were dried and their weights were measured to calculate the microbial activity. The sulfate reduction rates were calculated from the rates of decrease in sulfate in Exps. S1 and S2. The denitrification rates were calculated from the rates of decrease in nitrate in Exps. N1 and N2. The sulfur denitrification rates were calculated from the rates of sulfate increase in Exps. TN1 and TN2.

**Table 2:** The experimental conditions used in the batch experiments.

Exp. no.	B1	B2	S1	S2
Wood in the reactor (g-wet weight/90mL)				
Cedar	25	-	25	-
Aspen	-	25	-	25
The added substrates (mg/L)				
K_2_SO_4_	-	-	181	181
NaNO_3_	-	-	-	-
Na_2_S_2_O_3_∙5H_2_O	-	-	-	-
Exp. no.	N1	N2	TN1	TN2
Wood in the reactor(g-wet weight/90mL)				
Cedar	25	-	25	-
Aspen	-	25	-	25
The added substrates (mg/L)				
K_2_SO_4_	-	-	-	-
NaNO_3_	137	137	137	137
Na_2_S_2_O_3_∙5H_2_O	-	-	250	250

### 2.3. Analytical Methods

We determined the concentrations of TOC and TN with a TOC analyser (TOC-V CPH/CPN; Shimadzu Co., Kyoto, Japan). Total phosphorus (TP) and PO_4_^3−^-P were determined by a nutrient analyser (AACS III; Bran + Luebbe, Norderstedt, Germany). The concentration of organic acids was determined by ion chromatography (LC-10AD; Shimadzu). The concentrations of sulfate, nitrite, nitrate and ammonium ions were determined using another ion chromatography system (IC-10AD; Shimadzu). The pH values were obtained using a glass electrode (pH/ION Meter F-24; Horiba, Kyoto, Japan).

### 2.4. Dissimilatory (bi)sulfite Reductase Gene-Targeted Nested PCR-DGGE Analysis

We examined the microbial communities of sulfate-reducing bacteria in the biofilm inside the wood by dissimilatory (bi)sulfite reductase gene-targeted nested PCR-DGGE. After 426, 749, 1104 and 1224 days of operation, we sliced the surface of the wood in Reactors 2-1 and 2-2 to a depth of approximately 5 mm, and we extracted DNA using the UltraClean Soil DNA kit (MoBio, Solana Beach, CA, USA). The *dsrB* fragments of extracted DNA were amplified with a two-step nested PCR protocol using combinations of primers targeting the dissimilatory (bi)sulfite reductase genes, and the DGGE analysis was performed as described previously [4].

To identify DGGE bands, we excised a small piece of gel from the middle of the target band using a gel chip (Funa gel chip; Funakoshi Co., Tokyo, Japan) and incubated it in 20 μL of distilled water for 24 h at 4 °C. The eluted DNA was reamplified by PCR using the primers DSRp2060F (5’-CAACATCGTYCAYACCCAGGG-3’) and DSR4R (5’-GTGTAGCAGTTACCGCA-3’) without a GC clamp [11]. The PCR products for sequencing were purified using a GenElute PCR Clean-Up Kit (Sigma-Aldrich, St. Louis, MO, USA) and sequenced using a DYEnamic ET Terminator Cycle Sequencing Kit and an ABI PRISM 3100 Genetic Analyzer (Applied Biosystems Japan, Tokyo, Japan).

To determine the closest known relatives of the partial DNA sequences obtained, we performed searches in the public data library DDBJ using FASTA and BLAST search tools [12]. All *dsrB* protein sequences were deposited in the database under the accession numbers AB808659–AB808680.

**Figure 2:**
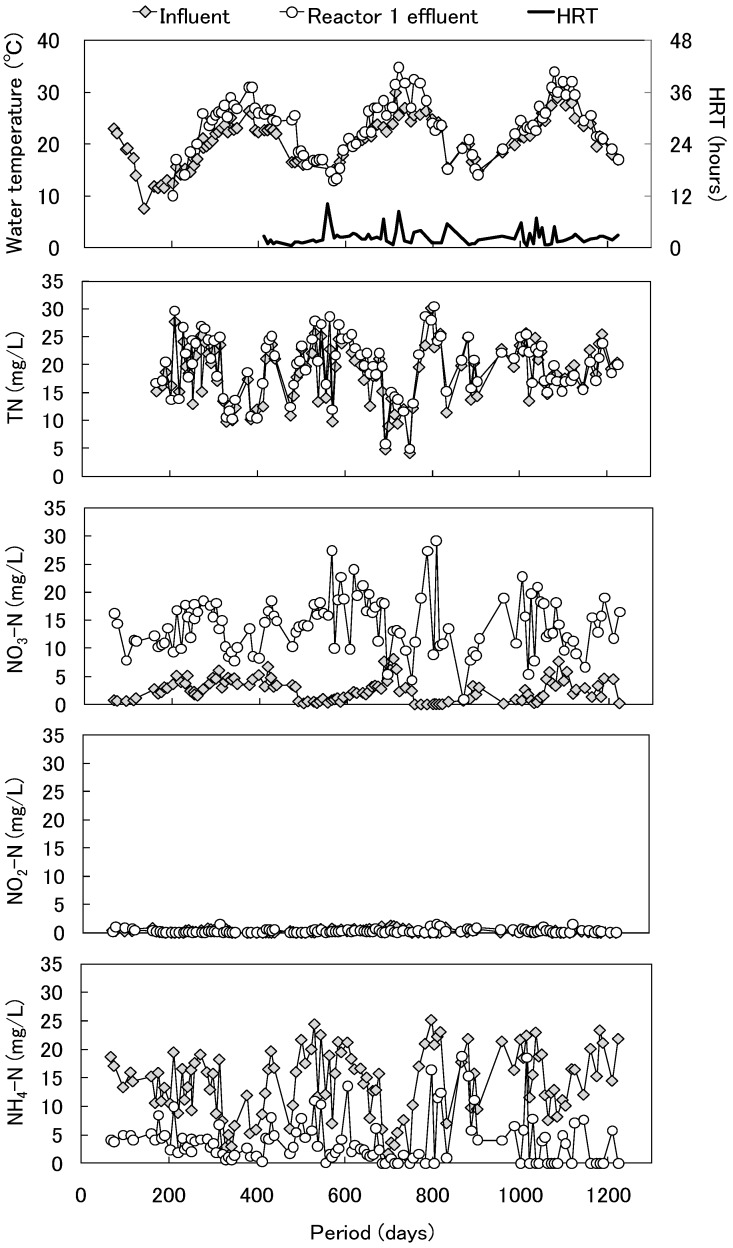
Time courses of water temperature and HRT, and the concentrations of TN, NO_3_-N, NO_2_-N, and NH_4_-N in Reactor 1.

## 3. Results and Discussion

### 3.1. Nitrification Performance of the Trickling Filter Packed with foam Ceramics (Reactor 1)

The time courses of the concentrations of TN, NO_3_-N, NO_2_-N and NH_4_-N and the water temperature in Reactor 1 are shown in Figure 2. The water temperature was 10°C to 35 °C (mean, 23 °C) during the operational period. The TN concentration was not changed by the reactor. The ammonium concentration decreased and the nitrate concentration increased. No accumulation of NO_2_-N was observed during the operational period. The ammonia oxidation occurred after 23 days of operation. These results indicate that the start-up of Reactor 1 was comparatively short for the application of foam ceramics as a microbial supporting medium.

**Figure 3:**
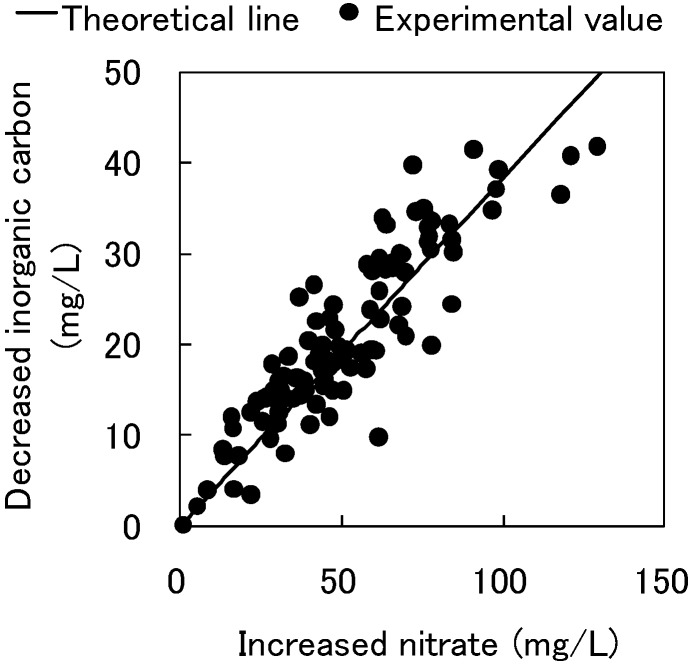
The relationship between increased nitrate and decreased inorganic carbon in Reactor 1.

The relationship between the decreased NH_4_-N and increased NO_3_-N in Reactor 1 is shown in Figure S1 of the Supplementary Materials. The decreased NH_4_-N was associated with an increase in NO_3_-N, indicating that nitrification occurred steadily in Reactor 1. In addition, NO_3_-N accumulated without the occurrence of denitrification. We suspected that the influent of Reactor 1 contained almost no organic compounds as electron donors for denitrification. The relationship between the increased nitrate and decreased inorganic carbon in Reactor 1 is shown in Figure 3. Based on the assumption that the gross composition of *Nitrosomonas* and *Nitrobacter* can be represented by C_5_H_7_NO_2_, the nitrification can be described by the following equation [13]:

NH_4_^+^ + 1.83O_2_ + 1.98HCO_3_^−^ → 0.98NO_3_^−^ + 0.021C_5_H_7_NO_2_ + 1.041H_2_O + 1.88H_2_CO_3_ (1)

The pH values of the influent and effluent of Reactor 1 were almost the same: 7.0 ± 0.3 and 7.0 ± 0.4, respectively. We assumed that the neutral pH was stable because the effluent from the final sedimentation basin contains an adequate amount of carbonate, even if the nitrifying bacteria consumed carbonate (Equation (1)).

**Figure 4:**
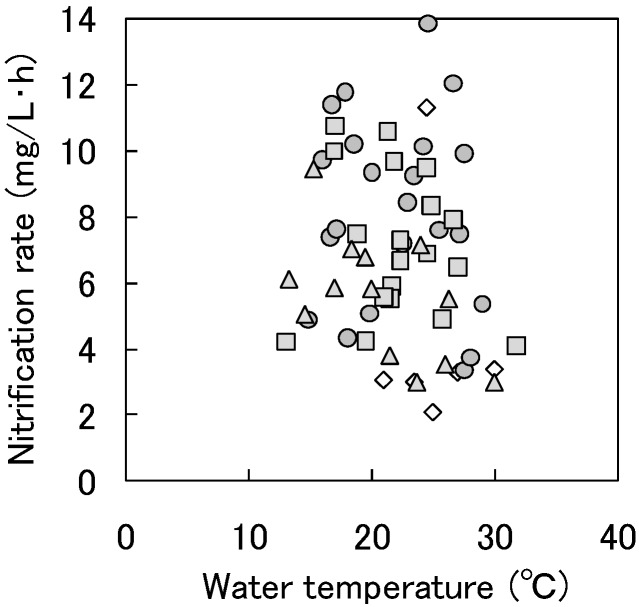
The relationship between the water temperature and the nitrification rate in Reactor 1.

The relationship between the water temperature and the nitrification rate in Reactor 1 is shown in Figure 4. The average nitrification rate in the trickling filter was 6.9 mg/L∙h (0.17 kg N/m^3^∙day) and stayed at 4.5 mg/L∙h (0.11 kg N/m^3^∙day) even when the water temperature was below 15 °C. The recycle ratio had relatively little effect on the nitrification rate and the nitrification rate did not tend to increase with increasing water temperature.

As reported previously, the maximum steady-state ammonia removal rate was 0.25 kg N/m^3^∙day in a continuous stirred-tank hollow-fiber membrane biofilm reactor using synthetic wastewater [14]. In an aerobic upflow fluidized bed reactor using granulation of nitrifying bacteria, the maximum ammonia removal rate was 1.5 kg-N/m^3^/day in a nitrification process for inorganic wastewater (containing 500 g/m^3^ of NH_4_^+^-N) [15]. In an airlift reactor with nitrifiers immobilized in polyvinyl alcohol for a marine recirculating aquarium system, the maximum ammonia removal rate was 0.07 kg-N/m^3^/day [16]. The apparent nitrification rate was approximately 0.18 kg-N/m^3^/day in nitrifying trickling filters at the Littleton/Englewood wastewater treatment plant [17].

In the present study, the average nitrification rate of the trickling filter was 0.17 kg N/m^3^∙day. This ammonia removal rate was comparable to the rates reported above. The trickling filter packed with foam ceramics has advantages in that it is a simple structure for nitrification and has lower operation and maintenance costs than aeration systems.

### 3.2. Nitrogen and Phosphorus Removal in the Anoxic Bioreactor Packed with Wood and Iron (Reactors 2-1 and 2-2)

**Figure 5:**
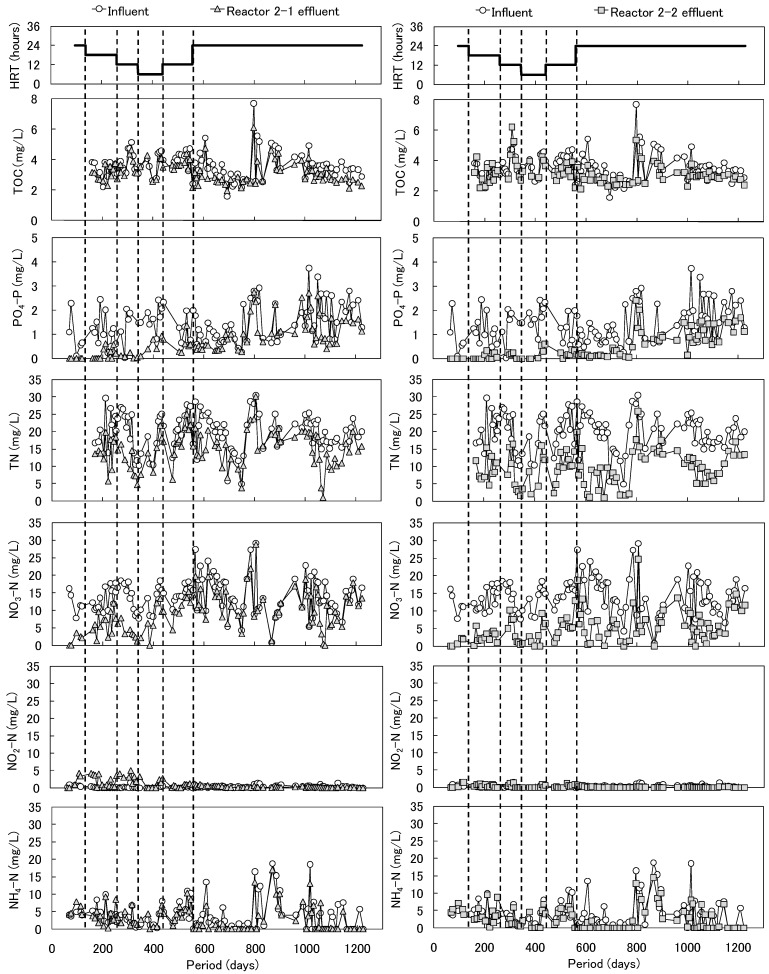
Time courses of HRT and the concentrations of TOC, PO_4_-P, TN, NO_3_-N, NO_2_-N, and NH_4_-N in Reactors 2-1 and 2-2.

The courses of the HRT and the concentrations of TOC, PO_4_-P, TN, NO_3_-N, NO_2_-N, and NH_4_-N in Reactors 2-1 and 2-2 are shown in Figure 5. The concentration of PO_4_-P in the influent was 0.1 to 2.4 mg/L. Until 343 days of operation, most of the PO_4_-P in the influent was removed from both reactors, although the HRT was shortened from 24 to 12 h. After 343 days of operation, the PO_4_-P removal in Reactor 2-1 (packed with cedar chips and iron) was lower than that of Reactor 2-2 (packed with aspen-wood chopsticks and iron). The removal performance of Reactor 2-2 continued over 1200 days of operation, although the phosphorus removal rate showed a gradual decline.

Using artificial wastewater, we previously examined the nitrogen and phosphorus removal in a biological filter reactor packed with iron and wood, and we found that PO_4_-P could be removed with this set-up, since ferrous ions were released from the iron by hydrogenotrophic denitrification and combined with phosphate [10]. The decrease in the PO_4_-P removal rate observed in the present study would have been due to excessive iron corrosion, in which an excessive amount of ferrous ions were released, formed iron oxide and attached to the surface of the iron. On the other hand, NO_3_-N decreased and NO_2_-N accumulated in Reactor 2-1 from 259 to 350 days of operation, and in Reactor 2-2, NO_3_-N decreased without accumulating NO_2_-N. Excessive concentrations of nitrate or nitrite in drinking water can cause methaemoglobinaemia.

The relative potency for nitrite with respect to methaemoglobin formation is more than 10 times higher than that for nitrate (on a molar basis), and the World Health Organization (WHO) guidelines for drinking water quality include a provisional value of 3 mg/L for nitrite [18]. Effluent nitrite in a reactor would thus be unfavorable. From a safety standpoint, we suspected that aspen wood would be a better carbon source than cedar chips, and we found that the TOC level in Reactor 2-1 (packed with cedar chips and iron) decreased to a mean value of 0.9 mg/L and that the TOC level in Reactor 2-2 (packed with aspen wood and iron) decreased to a mean of 0.8 mg/L. It was thus confirmed that there was no increase in the amount of dissolved organic matter in the outflow from the bioreactors. The biological treatment of wastewater with no organic compounds would therefore benefit from the use of wood as the carbon source. The pH of the influent in Reactors 2-1 and 2-2 was 7.0 ± 0.3. The pH of the effluent in Reactor 2-1 was 8.4 ± 0.5 until 378 days of operation and then 7.2 ± 0.4. The pH of the effluent in Reactor 2-2 did not increase until 378 days of operation and was 7.2 ± 0.3 during the entire operational period (data not shown). The pH increase in Reactor 2-1 might cause hydrogen-ion consumption by hydrogenotrophic bacteria, but further investigations are required to confidently make this assertion.

The high seasonal variations of nitrogen and phosphorus concentrations in the effluent from the STP had an effect on the effluent in Reactors 2-1 and 2-2, and thus the removal efficiency of nitrogen and phosphorus was unclear. The time courses of PO_4_-P removal efficiency per HRT, the nitrogen removal efficiency per HRT, and the water temperature in Reactors 2-1 and 2-2 were determined (see Figure S2 of the Supplementary Materials). The water temperature was 7 °C to 38 °C (mean, 21 °C) during the operational period. The water temperature variation showed the same tendency as that of Reactor 1; the mean temperature difference between Reactor 1 and Reactors 2-1 and 2-2 was 2 °C. The variation of the removal efficiency per HRT showed the same tendency between phosphorus and nitrogen and was temperature-dependent. We found previously that nitrogen removal correlated better with phosphorus removal in a denitrification reactor packed with iron and wood treating artificial wastewater [10]. In the present study, the nitrogen removal efficiency decreased and the phosphorus removal efficiency also decreased in the cold season.

The nitrogen and phosphorus removal in Reactor 2-1 continued until about 500 days of operation, but the removal decreased significantly after that period. In contrast, the nitrogen and phosphorus removal in Reactor 2-2 continued throughout the operational period, although the removal decreased during the cold season. The removal performance of Reactor 2-2 packed with aspen wood and iron was higher than that of Reactor 2-1 packed with cedar chips and iron during the entire operational period.

**Figure 6:**
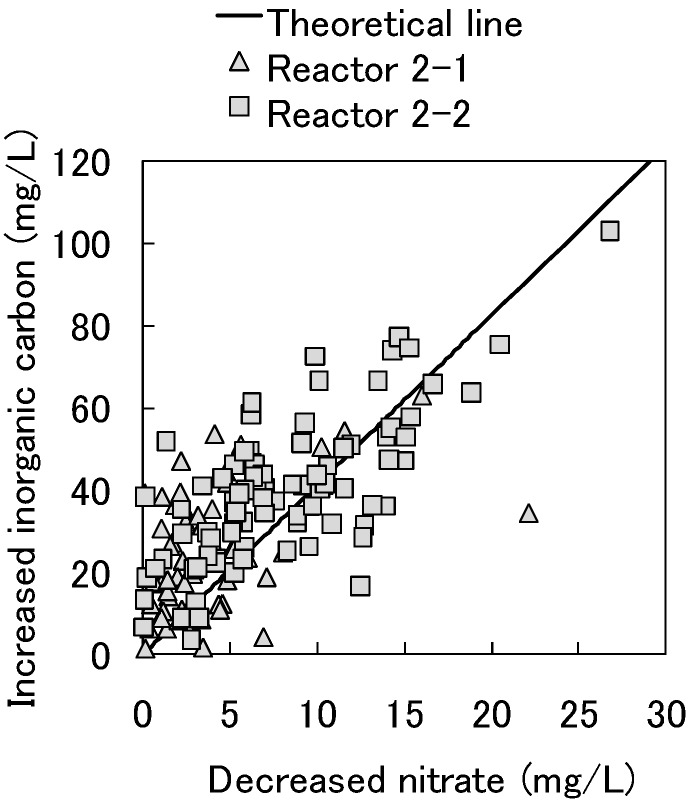
The relationship between decreased nitrate and increased inorganic carbon in Reactors 2-1 and 2-2.

The relationship between decreased nitrate and increased inorganic carbon in Reactors 2-1 and 2-2 is shown in Figure 6. The amounts of decreased nitrate correlated well with the amounts of increased inorganic carbon, and we thus speculated that the denitrification occurred via the cedar chips’ or aspen wood’s degradation. Cellulose degradation by denitrification can be described as shown below in Equation (2):

*NO*_3_^−^ + *H*^+^ + 1.25*CH*_2_*O* → 0.5*N*_2_ + 1.75*H*_2_*O* + 1.25*CO*_2_ (2)

In Equation (2), 1 mol (62 g) of nitrate reduction produces 1.25 mol (15 g) of inorganic carbon. The relationship between decreased nitrate and increased inorganic carbon in Figure 6 is in near complete agreement with the stoichiometric quantity, indicating cellulose degradation by denitrification.

### 3.3. Denitrification and Sulfate Reduction Activities in the Batch Experiments (Reactors 2-1 and 2-2)

**Figure 7:**
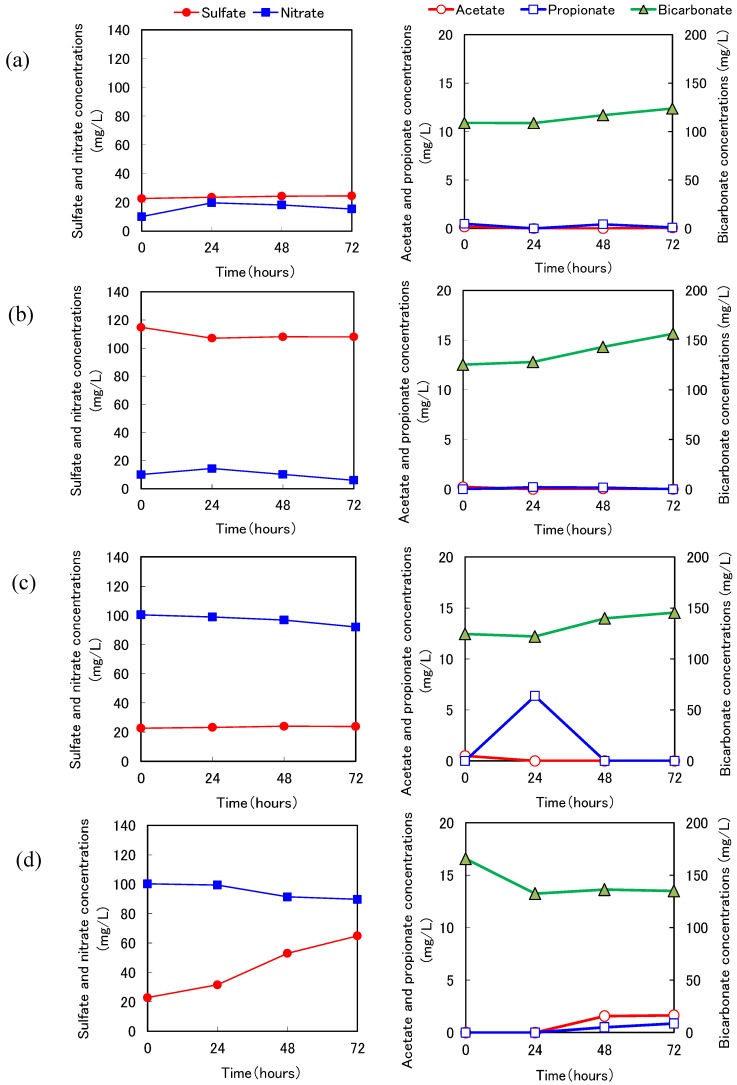
Typical results of batch experiments using the cedar chips taken from Reactor 2-1. (**a**) Exp. B1 was conducted under anaerobic conditions. (**b**) Exp. S1 was conducted under sulfidogenic conditions. (**c**) Exp. N1 was conducted under anoxic (denitrification) conditions. (**d**) Exp. TN1 was conducted under anoxic (sulfur denitrification) conditions (see Table 2).

**Figure 8:**
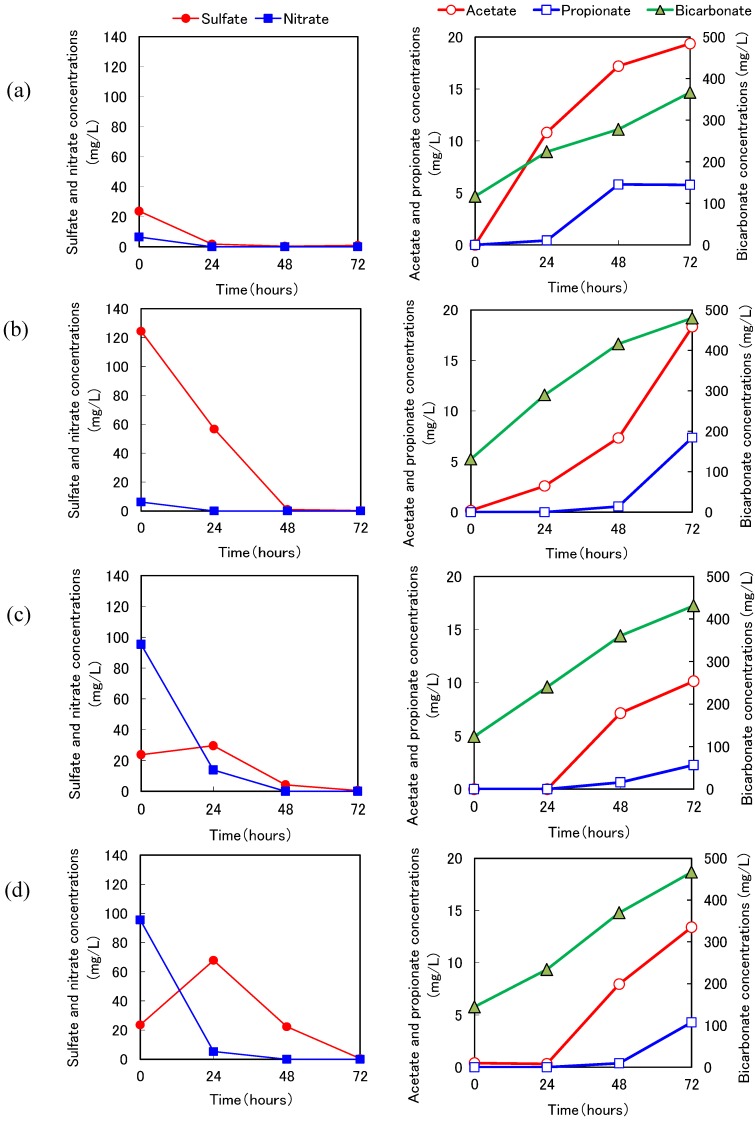
Typical results of batch experiments using the aspen wood taken from Reactor 2-2. (**a**) Exp. B2 was conducted under anaerobic conditions. (**b**) Exp. S2 was conducted under sulfidogenic conditions. (**c**) Exp. N2 was conducted under anoxic (denitrification) conditions. (**d**) Exp. TN2 was conducted under anoxic (sulfur denitrification) conditions (see Table 2).

Typical results of the batch experiments using wood taken from Reactors 2-1 and 2-2 at 593 days are shown in Figure 7 and Figure 8, respectively. In Exp. S1 and Exp. S2 under sulfidogenic conditions, sulfate decreased and inorganic carbon increased, indicating that microbes grew inside the wood and decomposed the wood under sulfidogenic conditions. The sulfate reduction activity was especially high when we used aspen wood taken from Reactor 2-2. Interestingly, propionate accumulated after the sulfate disappeared. The sulfate-reducing bacteria might have used propionate as an electron donor via wood degradation (Exp. S2, Figure 8).

We calculated the sulfate reduction rates from the rates of decrease in sulfate. In Exp. N1 and Exp. N2, under denitrification conditions, nitrate decreased and the increase in bicarbonate was higher than that in Exp. B1 and Exp. B2. These results indicated that heterotrophic denitrification with wood degradation occurred (Figure 7(c) and Figure 8(c)). In addition, acetate accumulated after the nitrate disappeared. Denitrifying bacteria might have used acetate as an electron donor. We speculate that the sulfate-reducing bacteria oxidized propionate to acetate and subsequently the denitrifying bacteria oxidized acetate to CO_2_ without competition, and the sulfate-reducing bacteria and denitrifying bacteria were segregated in the wood. The denitrification rates were thus calculated from the rates of decrease in nitrate.

In Exp. TN1 and Exp. TN2, under denitrification conditions using thiosulfate, the nitrate decreased and the sulfate increased. Moreover, in Exp. TN2 the sulfate decreased after the nitrate disappeared but not in association with acetate oxidation. These results suggested that sulfate-reducing bacteria inside the aspen wood grew without acetate as the carbon source. The sulfur denitrification rates were calculated from the rates of increase in sulfate. The concentrations of nitrite in all batch experiments were less than 3 mg/L and thus considered negligible (data not shown).

Table 3 shows the denitrification rates, sulfate reduction rates, and sulfur denitrification rates obtained from the batch experiments during the operational period under stationary conditions. The denitrification rates obtained with aspen wood were approximately 10 times higher than those obtained with cedar chips. Aspen wood does not have the antimicrobial properties associated with polyphenol, quinone and troponoid compounds because these compounds are not present in aspen wood. However, they are present in cedar chips, and thus we speculate that the difference in denitrification rates was due to the differing antimicrobial and other characteristics between cedar chips and aspen wood. The denitrification rates obtained with the aspen wood taken from Reactor 2-2 at 784 days was the highest in the batch experiments, at 0.42 g NO_3_-N/kg dry weight wood day. In an earlier study, the denitrification rate was 0.066 g N/kg wood chips day in a biofilter that used wood chips to treat subsurface drainage water [19].

**Table 3:** Denitrification and sulfate reduction rates obtained with the wood taken from Reactors 2-1 and 2-2 during the operational period.

	Denitrification Rates		Sulfur Denitrification Rates		Sulfate Reduction Rates	
	(g COD/kg dry weight wood∙day)		(g COD/kg dry weight wood∙day)		(g COD/kg dry weight wood∙day)	
Elapsed time (day)	Reactor 2-1 ^*^	Reactor 2-2 ^**^	Reactor 2-1 ^*^	Reactor 2-2 ^**^	Reactor 2-1 ^*^	Reactor 2-2 ^**^
593	0.03	0.92	0.12	0.44	0.02	0.82
784	0.08	1.21	0.04	0.91	0.04	0.53
1104	0.09	0.91	0.04	0.76	0.04	0.32
1224	0.10	0.85	0.01	0.46	0.07	0.25

^*^ The experiment used the cedar chips taken from Reactor 2-1. ^**^ The experiment used the aspen wood taken from Reactor 2-2.

The denitrification rates of the bioreactor packed with aspen wood in the present study were relatively high. The sulfate reduction rates and sulfur denitrification rates using the aspen wood taken from Reactor 2-2 were also approximately 10-fold higher than those using cedar chips taken from Reactor 2-1. This indicates that, compared to the organic compounds in cedar chips, the organic compounds in aspen wood are easier for sulfate-reducing bacteria and denitrifying bacteria to adapt for wood degradation. The high sulfur denitrification rates were involved in the high activities of sulfate reduction. Sulfate-reducing bacteria coexisting with sulfur-denitrifying bacteria and a sulfur oxidation–reduction cycle were established in Reactors 2-1 and 2-2.

In light of our finding that the denitrification activity of Reactors 2-1 and 2-2 lasted over 1200 days of operation, it is apparent that a reactor packed with cedar chips or aspen wood has advantages for denitrification in the long-term treatment of influent containing no organic compounds. We speculate that long-term denitrification could occur to degrade wood gradually in such a reactor.

### 3.4. Microbial Communities of Sulfate-Reducing Bacteria Attached to the Wood and Iron Taken from Reactors 2-1 and 2-2

We used *dsrB* gene-targeted nested PCR-DGGE to examine the microbial communities of sulfate-reducing bacteria inside the wood taken from Reactors 2-1 and 2-2 after 426, 749, 1104 and 1224 days of operation. Table 4 shows the closest microbial matches of the sequenced *dsrB* gene DGGE bands. The detected *dsrB* gene DGGE bands from the cedar chips taken from Reactor 2-1 were 1-1 to 1-11, and the DGGE bands from the aspen wood taken from Reactor 2-2 were 2-1 to 2-11. A *dsrA* or *dsrB*-protein homology greater than 75% would be considered to indicate inclusion in the same genus, in loosely related genera, or in the same family [20].

In the present study, the following bands were most closely related to the respective species: 1-2, *Desulfobulbus propionicus* (80.6% identity); 1-3, *Desulfoarculus baarsii* (76.9% identity); 1-5, *Desulfoarculus baarsii* (79.1% identity); 1-6, *Desulfovibrio fructosovorans* (75.4% identity); 1-9, *Desulfovibrio carbinolicus* (79.8% identity); and 1-10, *Desulfovibrio aminophilus* (76.6% identity). The microbial communities of sulfate-reducing bacteria in Reactor 2-1 changed during the long-term operation. The genus *Desulfobulbus* can use propionate, lactate, pyruvate, ethanol and others as electron donors and also as carbon sources, and organic compounds are incompletely oxidized to acetate [21]. The genus *Desulfoarculus* can use formate, butyrate, and higher fatty acids as electron donors and carbon sources, and these compounds are completely oxidized to CO_2_ [22]. Most of the species of the genus *Desulfovibrio* can oxidize organic compounds such as lactate incompletely to acetate, which cannot be oxidized further [23].

These results showed that sulfate-reducing bacteria growing within cedar wood mixed the completely and incompletely oxidized organic compounds. In previous research using artificial wastewater in a denitrification reactor packed with cedar wood, the sulfate-reducing bacteria detected were of the genera *Desulfobulbus* and *Desulfomicrobium* [4]. Among the sulfate-reducing bacteria, the genus *Desulfobulbus* might grow easily inside cedar wood. In the biofilm of aspen wood taken from Reactor 2-2, the following bands were most closely related to the respective species: 2-2, *Desulfobulbus rhabdoformis* (86.7% identity); 2-4, *Desulfacinum infernum* (78.1% identity); 2-5, *Desulfovibrio fructosovorans* (77.0% identity); 2-6, *Syntrophobacter fumaroxidans* (81.4% identity); 2-7, *Desulfofustis glycolicus* (82.3% identity); and 2-8, *Desulfovibrio aerotolerans* (77.9% identity).

The microbial communities of sulfate-reducing bacteria in Reactor 2-2 also changed during the long-term operation. This finding agrees with the result obtained from the batch experiment under sulfidogenic conditions (Exp. S2, Figure 8), in which propionate accumulated after sulfate disappeared. This implies that the genus *Desulfobulbus* would use propionate via wood degradation in Reactor 2-2. Interestingly, the genus *Desulfobulbus* was detected only at 426 days, whereas the genus *Desulfovibrio* was frequently detected during the operational period in this study. These sulfate-reducing bacteria are known to oxidize organic compounds incompletely, and thus denitrifying bacteria would use mainly acetate, because acetate was not detected under the remaining nitrate conditions in the batch experiments (Exps. N2 and TN2, Figure 8).

**Table 4:** Closest microbial match of sequenced *dsr B* gene DGGE bands.

Reactor No.	Elapsed Time (day)	Band ^a^	FASTA					BLAST		
			Sequencelength (bp)	Closest Relative	Accession No.^b^	% Identity ^c^	Overlap (bp)	Closest Relative	Accession No. ^d^	% Identity^c^
2-1	426	1-1	321	Desulfovibrio vulgaris	AE017285	64.0	314	Desulfovibrio vulgaris	AE017285	93(31/33)
		1-2	320	Desulfobulbus propionicus	AF218452	80.6	319	Desulfobulbus propionicus	AF218452	84(144/170)
	749	1-3	340	Desulfoarculus baarsii	AF334600	76.9	325	Desulfoarculus baarsii	AF334600	89(130/145)
		1-4	335	Desulfovibrio fructosovorans	AF418187	72.0	311	Desulfovibrio sp.	AF360650	90(72/80)
	1104	1-5	280	Desulfoarculus baarsii	AF334600	79.1	268	Desulfoarculus baarsii	AF334600	89(128/143)
		1-6	290	Desulfovibrio fructosovorans	AF418187	75.4	264	Desulfovibrio sp	AF360650	92(62/67)
	1224	1-7	309	Desulfotomaculum thermocisternum	AF074396	63.1	314	Desulfotomaculum reducens	CP000612	100(26/26)
		1-8	311	Desulfotomaculum putei dissimilato	AF273032	70.3	290	Desulfotomaculum partial	FM999736	94(34/36)
		1-9	316	Desulfovibrio carbinolicus	AY626026	79.8	267	Desulfovibrio carbinolicus	AY626026	92(95/103)
		1-10	309	Desulfovibrio aminophilus	AY626029	76.6	308	Desulfovibrio sp.	U58117	83(136/162)
		1-11	298	Desulfovibrio aespoeensis	AF492838	65.5	258	Desulfovibrio sp.	AF360650	90(37/41)
2-2	426	2-1	359	Desulfobulbus elongatus	AJ310430	73.0	337	Desulfobulbus elongatus	AJ310430	80(161/200)
		2-2	349	Desulfobulbus rhabdoformis	AJ250473	86.7	330	Desulfobulbus rhabdoformis	AJ250473	90(228/253)
		2-3	363	Desulfovibrio gigas	U80961	72.4	348	Desulfovibrio gigas	U80961	82(149/180)
	749	2-4	280	Desulfacinum infernum	AF418194	78.1	274	Desulfacinum infernum	AF482454	93(73/78)
		2-5	287	Desulfovibrio fructosovorans	AB061538	77.0	261	Desulfovibrio sp	AF360650	91(75/82)
		2-6	243	Syntrophobacter fumaroxidans	CP000478	81.4	242	Syntrophobacter fumaroxidans	CP000478	89(106/119)
	1104	2-7	290	Desulfofustis glycolicus	AF418191	82.3	282	Desulfofustis glycolicus	AF482457	83(212/253)
		2-8	290	Desulfovibrio aerotolerans	AY749039	77.9	281	Desulfovibrio aminophilus	AY626029	92(78/84)
	1224	2-9	311	Desulfofustis glycolicus	AF418191	73.9	264	Desulfofustis glycolicus	AF482457	82(114/139)
		2-10	263	Syntrophobacter fumaroxidans	CP000478	73.7	255	Syntrophobacter fumaroxidans	CP000478	85(61/71)
		2-11	329	Desulfovibrio carbinolicus	AY626026	69.6	313	Desulfovibrio sp.	AF360650	90(63/70)

^a^ Sequence analysis of bands excised from DGGE gels derived from bacterial 16S rRNA extracted from the wood in Reactors 2-1 and 2-2. ^b^ Accession number of sequence of the closest relative found by FASTA search. ^c^ Percentage of identical nucleotides in the sequence obtained from the DGGE band and the sequence of the closest relative found in the GenBank database. ^d^ Accession number of sequence of the closest relative found by BLAST search.

The genus *Desulfacinum* can use a wide range of organic acids and alcohols as electron donors and completely oxidizes them to CO_2_ [24]. The genus *Syntrophobacter* cannot oxidize acetate or fatty acids except for propionate [22,25]. The genus *Desulfofustis* can utilize organic substrates oxidized incompletely to acetate and glycolate or glyoxylate completely oxidized to CO_2_ [26]. In the present study it was unclear whether these sulfate-reducing bacteria were the dominant species in the wood, but we found that the species grew inside the wood under denitrification conditions in the presence of treated sewage in a long-term operation.

## 4. Conclusions

We evaluated the removal of nitrogen and phosphorus from the effluent of a sewage treatment, in an anoxic bioreactor over a long-term operation. We examined the nitrification ability of a bioreactor packed with foam ceramics and the nitrogen and phosphorus removal abilities of two bioreactors packed with wood and iron as electron donors. The results can be summarized as follows.

1. The average nitrification rate in the trickling filter was 0.17 kg N/m^3^∙day and stayed at 0.11 kg N/m^3^∙day even when the water temperature was below 15 °C. The recycle ratio had relatively little effect on the nitrification rate and the nitrification rate did not tend to increase with increasing water temperature.
2. The nitrogen and phosphorus removal in the bioreactor packed with cedar chips and iron continued until approximately 500 days of operation, but the removal decreased significantly after that period. In contrast, the nitrogen and phosphorus removal in the bioreactor packed with aspen wood and iron continued over 1200 days of operation. The nitrogen and phosphorus removal performance of the bioreactor packed with aspen wood and iron was higher than that of the bioreactor packed with cedar chips and iron during the operational period.
3. The TOC in the bioreactor packed with wood and iron decreased to a mean value of 1 mg/L, and thus it was confirmed that there was no increase in the amount of dissolved organic matter in the outflow from the bioreactors.
4. The denitrification rate obtained using the aspen wood taken from Reactor 2-2 at 784 days was the highest of the batch experiments, at 0.42 g NO_3_-N/kg dry weight wood∙day.
5. Compared to the organic compounds in the cedar chips, the organic compounds in the aspen wood were easier for the sulfate-reducing bacteria and denitrifying bacteria to utilize for wood degradation.
6. Sulfate-reducing bacteria grew inside the wood in a long-term operation under denitrification conditions, although the microbial communities of sulfate-reducing bacteria in Reactors 2-1 and 2-2 changed during the operation.

## Supplementary Files

Supplementary File 1
