# Supplementary material for: Nitrogen and Phosphorus Removal from Wastewater Treatment Plant Effluent via Bacterial Sulfate Reduction in an Anoxic Bioreactor Packed with Wood and Iron
Source: Int J Environ Res Public Health. 2014 Sep 22;11(9):9835–53. doi: 10.3390/ijerph110909835 (PMC4199053; doi:10.3390/ijerph110909835)
Supplement: Supplementary File 1 [file ijerph-11-09835-s001.pdf]

Supplementary Information

Nitrogen and Phosphorus Removal from Wastewater Treatment Plant Effluent via Bacterial Sulfate Reduction in an Anoxic Bioreactor Packed with Wood and Iron

Figure S1. The relationship between decreased NH_4-N and increased NO_3-N in Reactor 1.

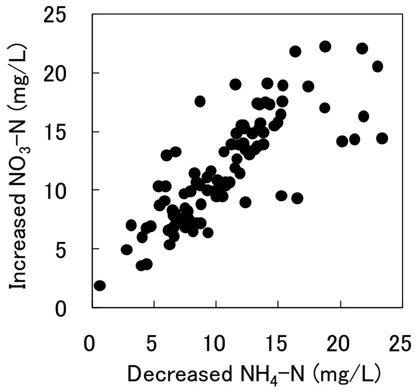

Figure S2. Time courses of PO_4-P removal efficiency per HRT, nitrogen removal efficiency per HRT and water temperature in Reactors 2-1 and 2-2.

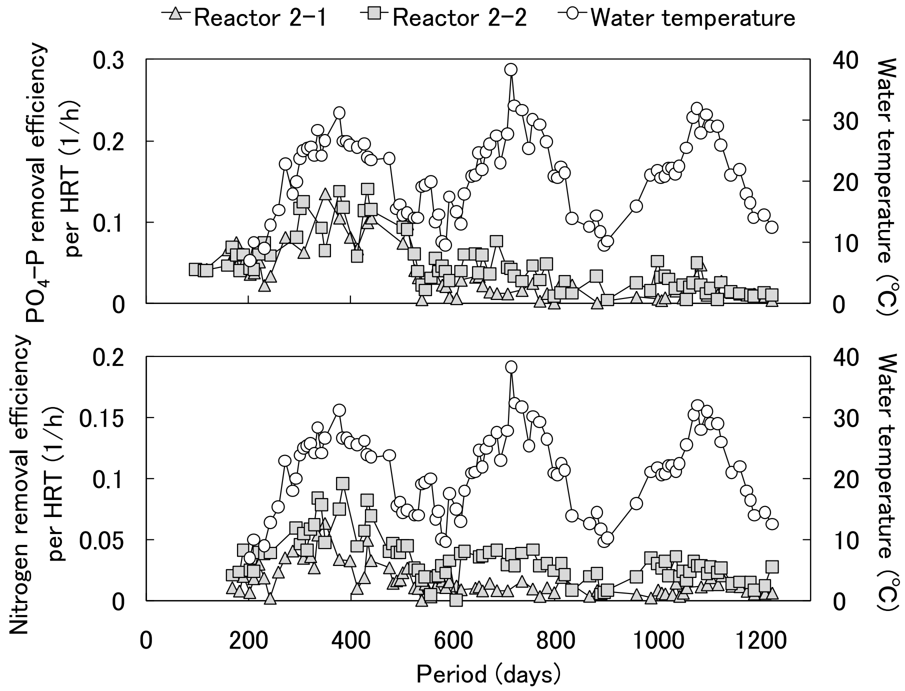

© 2014 by the authors; licensee MDPI, Basel, Switzerland. This article is an open access article distributed under the terms and conditions of the Creative Commons Attribution license (http://creativecommons.org/licenses/by/3.0/).
